# Patient Derived Organoids (PDOs), Extracellular Matrix (ECM), Tumor Microenvironment (TME) and Drug Screening: State of the Art and Clinical Implications of Ovarian Cancer Organoids in the Era of Precision Medicine

**DOI:** 10.3390/cancers15072059

**Published:** 2023-03-30

**Authors:** Giulia Spagnol, Francesca Sensi, Orazio De Tommasi, Matteo Marchetti, Giulio Bonaldo, Livia Xhindoli, Marco Noventa, Marco Agostini, Roberto Tozzi, Carlo Saccardi

**Affiliations:** 1 Department of Women and Children’s Health, Clinic of Gynecology and Obstetrics, University of Padua, 35100 Padua, Italy; 2 Department of Women and Children’s Health, University of Padua, 35100 Padua, Italy; 3 Fondazione Istituto di Ricerca Pediatrica Città della Speranza, 35129 Padua, Italy; 4 General Surgery 3, Department of Surgical, Oncological, and Gastroenterological Sciences, University of Padua, 35100 Padua, Italy

**Keywords:** ovarian cancer, organoids, tumor microenvironment, extracellular matrix, drug screening

## Abstract

**Simple Summary:**

Ovarian cancer (OC) has the highest mortality rate of any gynecological malignancy due to the advanced-stage diagnosis, high therapeutic resistance, high recurrence rate, and lack of targeted personalized treatments. This requires the development of preclinical models that can mimic the histological, molecular, and pathophysiological characteristics of various OC subtypes according to patient characteristics. In this scenario, patient-derived organoids represent an emerging model (PDOs). PDOs are 3D dynamic tumor models that can be grown successfully from patient-derived ovarian tumor tissue, ascites, or pleural effusion. This model recapitulates the heterogeneity of OC and allows for drug screening as well as the development of new target therapies. The purpose of this study is to provide information on PDOs and the critical role of the extracellular matrix (ECM) and the tumor microenvironment (TME) in their development to implement precision medicine in patients with patients with patients with ovarian cancer.

**Abstract:**

Ovarian cancer (OC) has the highest mortality rate of all gynecological malignancies due to the high prevalence of advanced stages of diagnosis and the high rate of recurrence. Furthermore, the heterogeneity of OC tumors contributes to the rapid development of resistance to conventional chemotherapy. In recent years, in order to overcome these problems, targeted therapies have been introduced in various types of tumors, including gynecological cancer. However, the lack of predictive biomarkers showing different clinical benefits limits the effectiveness of these therapies. This requires the development of preclinical models that can replicate the histological and molecular characteristics of OC subtypes. In this scenario, organoids become an important preclinical model for personalized medicine. In fact, patient-derived organoids (PDO) recapture tumor heterogeneity with the possibility of performing drug screening. However, to best reproduce the patient’s characteristics, it is necessary to develop a specific extracellular matrix (ECM) and introduce a tumor microenvironment (TME), which both represent an actual object of study to improve drug screening, particularly when used in targeted therapy and immunotherapy to guide therapeutic decisions. In this review, we summarize the current state of the art for the screening of PDOs, ECM, TME, and drugs in the setting of OC, as well as discussing the clinical implications and future perspectives for the research of OC organoids.

## 1. Introduction

Epithelial ovarian cancer (EOC) is the leading cause of death from gynecologic malignancies worldwide [1,2]. The high mortality rate is mainly due to the predominance of late-stage detection and the high rate of recurrence due to chemotherapy resistance. The disease is discovered in advanced stages in more than 80% of patients (International Federation of Gynecology and Obstetrics, FIGO, stages III and IV), resulting in poor survival outcomes [3,4]. With many histological variations, OC has been regarded as a highly heterogeneous disease. EOC can be divided into two subtypes: type I tumors that grow slowly with a distinct set of frequently mutated genes, including KRAS, BRAF, PTEN, and CTNNB1, and include low-grade serous carcinoma (LGSC), mucinous carcinoma (MC), endometrioid carcinoma (EC), clear-cell carcinoma (CCC), and type II tumors that progress rapidly with mutations in the TP53 (96%), BRCA1 and BRCA2 genes (20%) and include high-grade serous carcinoma (HGSC), and carcinosarcoma [5,6,7,8,9,10].

Unfortunately, pathobiology is poorly understood and, in the era of precision medicine, preclinical models that accurately recapitulate the biological characteristics of tumors in vivo have become fundamental. The animal model and, in particular, patient-derived xenografts (PDX) and genetically engineered mouse models (GEMM), are believed to mimic the histological features of the original tumors but also respond to drugs in a similar way to those in clinical settings [11,12,13].

Despite the potential usefulness of these two methods in cancer research, the success rates of both methods vary between cancer types or are not even mentioned [14], which means that alternative ways to maintain primary tumor cells in an efficient and robust manner are required [15]. In this scenario, emerging technology that attempts to reproduce an effective model for use in the functional assays of individual patient tumors is represented by patient-derived organoids (PDOs).

Organoids, defined as self-developing, three-dimensional, in vitro reconstructions of tissues, provide powerful tools to model human disease. In the literature, tumors of the colon, prostate, breast, pancreas, endometrium, and other solid tumors have been propagated with a high rate of success using PDO techniques [16,17,18,19,20,21].

The resulting PDOs closely resemble the original patient tumor in morphology, mutation profile, and gene expression patterns [22,23].

In fact, significant studies conducted in the last 2–3 years have highlighted the potential role of OC PDOs in reproducing many of the challenging characteristics of the original tumor in a reasonable time frame. [24].

Moreover, accumulating evidence indicates the possibility of obtaining personalized drug-based therapy. On this basis, it is possible that PDOs could be introduced as a clinical test to guide the selection of therapeutic treatments [25].

Essential for the growth and expansion of PDOs of stem cells is the extracellular matrix (ECM). Currently, the gold standard for three-dimensional (3D) cancer models is to use the commercially available Matrigel consisting of a secreted cocktail of ECM proteins, growth and signaling factors that mimic the stem cell niche [5,26].

Different studies of cancer, not only OC, showed that the modifications and accumulation of components of the ECM in the surrounding tumor area are responsible for cancer development and response to chemotherapy [27].

Another important component of tissue cancer is represented by the tumor microenvironment (TME), a rich mixture of malignant and non-malignant cells, such as fibroblasts, endothelial cells, and immune cells, embedded in the ECM. Therefore, TME affects tumor growth and undergoes changes in response to cancer progression, such as stiffening of the ECM, shifts in chemical signaling, and tumor angiogenesis.

One of the major limits of tumor PDO is the lack of diffuse reliable protocols to reproduce TME, and some efforts are underway to include stromal, immune, and vascular cells in the cultures [28,29].

The aim of our review was to recapitulate the literature on PDOs, ECM, TME, and drug screening in the context of OC. Finally, given the importance of translational medicine, we discuss the advantages, limitations, clinical implications, and future perspectives of OC PDO research.

## 2. Patient-Derived Organoids (PDOs)

Several studies have reported on different types of genomic characterization used to compare native tumor tissues and PDOs, demonstrating that OC PDOs capture tumor heterogeneity such as nuclear and cellular atypia, as well as biomarker expression [30,31,32,33,34,35,36,37,38,39,40,41,42,43,44,45,46] (see Table 1 for details).

In 2017, Pauli et al. developed 56 PDOs extrapolated from a variety of tumors, 2.5% of which were OC with a success rate of 100% [30]. They performed a whole-exome sequencing (WES) on a tumor-based organoid, and when they compared primary tumor-derived organoids to native tumor tissues, allele-specific copy number analysis (CNAs) of 1062 specific cancer genes revealed a median of 86% concordance in ploidy and genomic burden [30]. In the same period, Jabs et al. released nine PDOs (success rate 100%) to compare, in parallel, drug responses between 2D and organoid cultures of patient cells and possible associations with genomic alterations. The genomic analysis was performed by means of whole genomic sequencing (WGS) [31]. Following these studies, Hill et al. proposed an interesting short-term organoid culture with an 80–90% success rate for PDO creation. The study established 33 organoid cultures derived from various tumoral tissues of 22 patients. PDOs’ genome and corresponding tumors were analyzed by WES and the analyses of both somatic and germline mutations revealed a high degree of agreement (98.8% of mutations found in the organoids were also present in the tumor). The same high concordance was found for the genome-wide copy-number status and somatic variants for all tumor–organoids. Moreover, the authors used PDOs to assess DNA damage repair defects and their impact on immune-oncologic agents, potentially providing a tool for predicting patient therapy response (see Drug Screening section) [32].

Differently, two studies tested the heterogeneity by comparing the p53 staining pattern of PDOs and original tumors. Both show a high degree of concordance with populations of p53-positive and p53-negative cells, maintaining the original tumor’s heterogeneity [33,34]. In particular, Phan et al. created four PDOs with a 100% success rate but did not perform additional genomic characterization [33]. Instead, Nanki et al. released 28 primary organoid cultures obtained from 35 patients with a success rate of 80%. They obtained a targeted capture sequencing of 1053 cancer-related genes with a median 59.1% shared gene variants and high concordance in copy number variations (CNVs) [34].

Following these studies, Kopper et al. published an important study in 2019. The authors presented EOC long-term PDOs platform from tumor resection, and/or the drainage of ascites/pleural effusion, either before or after (neoadjuvant) chemotherapy [35]. They developed 56 PDOs with a success rate of growth of 65% from 32 different patients. This important study performed accurate OC tissue processing, genomic analysis, histology, single-cell WGS library preparation, RNA-seq analysis, drug screening and viability assays (see Drug Screening section). A comprehensive analysis demonstrates that OC organoids maintained tumors’ histological characteristics and genomic landscape (nuclear and cellular atypia, and biomarker expression, such as p53 and PAX8), remained highly similar at the gene expression, even after extended passaging, recapitulated OC hallmarks, such as somatic single-nucleotide variants (S-SNVs), CNVs and captured tumor heterogeneity [35]. Furthermore, using the recombination capacity test, PDOs were tested for homologous-recombination (HR) deficiency (HRD), confirming the data published by Hill et al. [32].

A similar genomic analysis was performed in another study by Maru et al., which reported the propagation of nine ovarian organoid cultures with an overall growth success rate of 90% and high concordance rate in somatic mutations. The authors discovered that the variant allele frequency (VAF) of TP53 and PTEN mutations was stereotypically enriched from 70% in tumors to nearly 100% in organoids, implying that the loss of heterozygosity and a point mutation in each gene was a founder mutation shared by most cancer cells in the tumor, which were enriched simply as epithelial cells in organoids [36].

Hoffmann et al. established 15 fallopian tube organoids (growth rate, 29%) from patients with advanced HGSC with PTEN, p53, and Rb knockdown. The triple knockdown (KD) gave rise to pro-carcinogenic phenotypes such as enhanced DNA double-strand breaks, increased phosphorylated Akt/cyclin E1 and atypical nuclei. PDOs formed invasive tumors that preserve the histological biomarkers and properties of the initial OC tissue. It was further demonstrated that a low-Wnt environment and active BMP signaling were required to maintain stemness in both the triple-KD fallopian tube organoids and OC PDOs [37].

Differently to others study, Sun et al. evaluated 10 tumor PDOs considering 4 cisplatin-sensitive and 6 cisplatin-resistant donors. The authors performed an RNA sequencing of organoids to compare the expression of chemosensitivity-related genes both in cisplatin-sensitive and cisplatin-resistant PDOs. The results showed that many important regulatory genes involved in cell senescence were decreased while those of several genes involved in glycolysis were significantly increased. Particularly, AURORA-A levels were enhanced whereas SOX8 and FOXK1 levels were markedly decreased [38]. Similarly, Wang et al. developed 10 pairs of cisplatin-sensitive and resistant ovarian cancer organoids derived from as many patients who underwent cytoreductive surgery aiming to analyze the role of FBN1 in the chemoresistance process in ovarian cancer. FBN1 expression was significantly enhanced in cisplatin-resistant ovarian cancer organoids, showing that FBN1 might be a relevant factor in the chemoresistance of ovarian cancer [39].

In 2020, Maenhoudt et al. established 12 organoid cultures of tissue from 27 patient-derived OC with an overall growth rate of 44%. They performed the WGS. Compared to other studies, the authors analyzed the expression of the ERBB2 and ERBB3 receptor family in more depth, finding it to be highly expressed in the organoids and the primary tumor, but the role and clinical significance of ERBB2 (HER2) and ERBB3 (HER3) in OC remain unclear and controversial. The genomic study revealed that most somatic copy-number alterations (S-CNAs) and the genetic alterations detected (i.e., 1638) in primary tumors were retained in the corresponding organoid lines [40]. Similarly, De Witte et al. characterized 36 organoids from 23 OC patients with WGS. PDOs resembled the tumors from which they were derived, finding an average overlap of 67% between single-nucleotide variants and similar CNAs profiles [41]. Another study was conducted by Chen et al. [42]. They generated 14 organoid cultures extrapolated from 6 patients with OC (3 organoid donors had neoadjuvant chemotherapy prior to specimen collection) obtained from serous effusions (both ascites and pleural effusions). The peculiarity was the characterization of organoids from multicellular spheroids MCS using formalin-fixed, paraffin-embedded H&E stain, Ki67 index evaluation, and genomic characterization using RNA-Seq analysis, which yielded 1584 differentially expressed genes [42]. Gorski et al. developed six tumor-based PDOs (success rate 1005) with the aim of analyzing chemosensitivity and investigating the genomic mediators of platinum resistance. Genomic sequencing was released, employing a Tempus xT gene panel and network mapping to explore the effect of carboplatin on genes (see Drug Screening section) [43].

Tao et al. derived PDOs with a success rate of 85% by performing WES and CNVs. Genome-wide CNVs analysis revealed similar patterns of DNA copy number losses and gains between organoid/tumor pairs. To further quantify the genetic correlation between PDOs and corresponding native tumors, the authors analyzed S-SNVs and structural variants (SVs). PDOs recapitulated the total mutational load and the different contributions of point mutation types of corresponding tumors with a 91.5% overlap [44].

More recently, Wan et al. released 13 tumor-based organoids derived from patients affected by HGSOC and 13 borderline ovarian tumor (BOT) organoids (with a success rate of 76.9%). Concordance between organoids and primary tumors was estimated by assessing the expression of diagnostic molecular markers such as ER, P53, Pan-CK, PAX8 and Ki67 [45,46].

## 3. Extracellular Matrix (ECM) and Tumor Microenvironment (TME)

Matrigel, derived from the murine Engelbreth–Holm–Swarm (EHS) sarcoma, is one of the most versatile ECMs for ovarian cancer cell in vitro modeling. It has been used as a basement membrane mimic and structural support for many cell types [47]. The major components of ECM are laminins, collagen IV, entactin, and the heparin sulfate proteoglycan [48]. Matrigel may also contain collagens I, XVIII, VI, and III, alongside growth factors and enzymes such as TGF-β, FGF, and matrix metalloproteinases (MMPs) [27,48]. Matrigel was used as a 3D ECM scaffold in the current studies, with cells cultured in a cocktail of growth and signaling factors (see Table 2 for details) [30,31,32,33,34,35,36,37,38,39,40,41,42,43,44,45,46]. Some authors described their personal cocktail of growth and signaling factors, with the establishment of OC PDOs in culture taking from 1 to 4 weeks. Hill et al. plated malignant cells in Matrigel and growth factor-enriched media with R-spondin-1 (RSPO1) but no estradiol for culture maintenance, and PDOs grew within 7–10 days [32]. Another study established a miniaturized approach to form a solid thin ring by plating tumoral cells pre-mixed with cold Matrigel around the rim of the wells [33]. This method, proposed by Phan et al., requires a small number of cells, and the ring configuration allows for the addition and removal of media by directly pipetting in the center of the well without damaging the gel [33]. Differently, Kopper et al. used a different type of support called Cultrex BME with two types of OC medium for organoid derivation: with (‘OCWNT medium’) or without (‘OC medium’) Wnt-conditioned medium [35]. The authors noted that the addition of hydrocortisone, forskolin and heregulinβ-1 improved the efficiency of OC PDO derivation [35]. Instead, Maru et al. modified a Matrigel bilayer organoid culture protocol (MBOC), introducing a seven-minute digestion step with Accumax, another potent proteolytic, and collagenolytic enzyme with DNase activity, following routine digestion [36]. Furthermore, undissolved cell aggregates were collected on day 1, when the upper layer of Matrigel was supposed to be overlaid, to undergo another round of Accumax treatment. These modifications to the original MBOC protocol resulted in an increase in growth rate from 45% to 83% [36]. Similarly, Maenhoudt et al. obtained an increase in the growth rate of PDOs by plating the cells in 70% growth-factor-reduced Matrigel/30% Dulbecco’s modified Eagle’s medium/F12, and cultured it in defined media [40]. The authors increased the expandability, number of organoids formed, and passage ability of OC PDOs by decreasing the concentration of the TGFb pathway inhibitor A83-01, increasing the level of nicotinamide, switching the source of RSPO1 from cell-line-conditioned medium to recombinant protein, and adding NRG1. OC PDOs typically developed within 2–4 weeks under these optimal conditions [40]. Differently, in the study of Wan et al., tumor cells were primarily dissociated by means of different additives (DMEM, FBS, penicillin-streptomycin, Type II collagenases). Therefore, obtained cells were then treated with IL-2 and conjugated with 15% Matrigel. In the end, they were plated and incubated with the above-mentioned drugs [45,46]. Instead, concerning the TME, only a few studies reconstructed the TME in vitro 3D, organotypic, co-culture models with two or more cell types for OC [28,49,50]. An omental mesothelium model was developed by Kenny et al. [49]. Taking a layered approach to reproduce the architecture of the omentum, as observed from the histological staining of omental biopsies, that this model is composed of primary human fibroblasts with ECM, rat-tail collagen-I and human fibronectin as a base, and layered with primary human mesothelial cells (HPMCs) isolated from fresh biopsies of the omentum. HPMCs were first overlaid on fibroblasts embedded in ECM. Fluorescently labelled OC cells were added next, and their adhesion and invasion were observed. Using this model, Kenny and colleagues identified the MMP-2-mediated cleavage of fibronectin and vitronectin produced by mesothelial cells as an early response to omental metastasis and a quantitative high-throughput screening (qHTS) assay was conducted to screen 44,420 structurally diverse small-molecule compounds that potentially inhibit OC metastasis [50].

## 4. Drug Screening

As underlined in the previous paragraph of our review, the OC PDOs recapitulated the main hallmarks of the original tumor, including CNVs, recurrent mutations, and tumor heterogeneity, while also providing the opportunity to test drug therapy [30,31,32,33,34,35,36,37,38,39,40,41,42,43,44,45,46]. In fact, the authors demonstrated that the drug–response curves revealed distinct sensitivities of the different PDO lines for the drugs, indicating patients’ tumor-dependent responses and highlighting the potential applicability of EOC-derived organoids as a drug screening platform [32,34,36,38,40]. Only some authors compared drug screening to clinical outcomes, specifically persistent disease or recurrence with clinical response and platinum resistance [33,34,38,41].

Drug screening using PDO technology can be carried out at an experimental research level or at an automated scale. Undoubtedly, the experiments that take place in research laboratories are based on a relatively limited number of replicates due to the small number of wells that can be obtained when a biopsy is processed. Although the PDO line can be expanded, laboratory screenings are always confined to a limited number and operator-related. Differently, in companies, drug screenings can take place in an automated way thanks to robotic technologies and the fact that companies can refer to different research centers or private entities by providing their PDO’ libraries, resulting in the production of more reproducible and robust data that are adequate for clinical trials (Phases I–III) [51]. Cytotoxicity is one of the main read-outs to be assessed in any drug screening investigation. Laboratory methods for assessing cell viability/death in organoids are similar to the traditional methods used in 2D cells, with modifications in terms of reagent concentrations or incubation times. In particular, the most commonly used methods are cell counting using dye exclusion methods; for example, trypan blue dye exclusion (i), staining for cell viability/cell death (ii) and metabolic activity testing (iii). All these methodologies are explained in detail in the review of Senem Kamiloglu, titled “Guidelines for cell viability assays” [52].

To date, the most widely used tests on PDOs use LIVE/DEAD™ Cell Imaging, which can discriminate between live and dead cells with two probes that measure recognized parameters of cytotoxicity and cell viability—intracellular esterase activity and plasma membrane integrity [53]. This test can reveal live and dead cells even if inside a support that mimics the ECM, such as Matrigel^®^ or Geltrex^®^. Another very useful kit is the TUNEL, used to detect DNA fragmentation, such as in an apoptosis cell state. Widely used in the laboratories are tests that evaluate the metabolic activity of PDO, such as MTT, WST-1, the Alamar Blue (AB) Resazurin, Alamar Blue, and CellTiter-Glo^®^. This class of methods uses the cellular metabolic activity to measure viability or proliferation in the PDO population. Metabolically active cells maintain a reducing environment within their cytosol.

This is taken advantage of through the use of colorimetric redox indicators and their conversion, which can be measured spectrophotometrically [54]. Typically, these tests are performed using different drug concentrations in order to obtain a dose–response curve and the half-maximal inhibitory concentration (IC50). Furthermore, in discovering synergistic drug combinations, the level of synergism is classically measured and quantified by the drug combination index (CI).

Most studies investigated the effect of chemotherapeutic agents commonly used in clinic to treat OC (i.e., paclitaxel, carboplatin, doxorubicin, and gemcitabine) on established PDOs [32,36,37,38,41]. On the other hand, some studies performed a drug screening using a specific drug panel including drugs targeting the PI3K/AKT/mTOR pathway (alpelisib, pictilisib, MK2206, AZD8055), poly (ADPribose) polymerase (PARP) inhibitors (PARPi) (Olaparib and Niraparib), the tyrosine kinase Wee1 (adavosertib), ReACp53, Staurosporine and protein kinase inhibitor compounds FDA-approved or in clinical development. Most HGSC PDOs showed higher sensitivity to platinum-based treatments, whereas PDOs from non-HGSOC (CCC, LGSC, EC) were more resistant; others were highly sensitive to gemcitabine, adavosertib, carboplatin and paclitaxel and resistant to drugs that target the PI3K/AKT/mTOR pathway (see Table 3 for details) [32,34,35,36,38,40,41,42].

Similarly, Gorski et al. discovered that comparing carboplatin-resistant PDOs to carboplatin-sensitive PDOs revealed an interplay of various patterns centered on the NF-KB pathway [43].

Further interesting results included the fact that Nutlin-3, a recently developed drug that targets TP53 wild-type OC, presented different sensitivities depending on the patient’s TP53 status. This finding was later replicated by OC PDOs, which were sensitive to nutlin-3 when derived from TP53 wild-type tumors but resistant when derived from TP53 mutant tumors [40]. Sun et al. demonstrated that tumors PDOs chemosensitivity assays were performed by administering cisplatin treatment on days 0 and day 21 with a significant increase in this family of mitotic serine/threonine kinases levels. The authors concluded that the analyses of drug-screening point out a pattern of mitotic serine/threonine kinases 6, AURORA-A, which could serve as a potential pharmacologic target to manage platinum-resistant tumors [38].

Furthermore, to overcome the technical constraints and extensive manipulations of screening in PDOs, Phan et al. established a miniaturized method by plating cells at the rim of the wells to form “mini-rings”. Using this platform, they successfully screened 240 kinase inhibitors on four OC organoids and identified personalized responses, underlining that a mini-rings approach is a reliable tool with the advantage of requiring a small number of cells without the need for expansion in vitro or in vivo [33].

One of the most important applications of drug screening in PDOs was the ability to test PARPi [31,32,34,35,44]. HRD cells have been shown to be sensitive to PARP inhibitors, and the HRD mutational signature of PDOs could predict sensitivity to PARPi treatment in both PDOs and the primary tumor [26]. Furthermore, OC PDOs were successfully used to directly test HR proficiency using biological assays, and this parameter was also consistent with the organoid’s sensitivity to PARP-inhibitors [35].

In Jabs et al., the HRD score was defined as the number of losses of heterozygosity regions observed in a tumor sample. These were correlated with cytotoxic responses to carboplatin and all its combinations and paclitaxel, azacytidine and decitabine responses. However, these drugs do not directly affect DNA structure or repair. Furthermore, positive HRD scores (≥10) determined for tumor tissues co-occurred with high drug-induced cytotoxicity. The authors demonstrated that the cytotoxicity triggered by BKM120 (PI3K inhibitor) and MK5108 (Aurora Kinase A inhibitor) was not altered by the amplification of AURKA and PI3KCA, respectively, and the loss of BRCA 1/2 did not affect the cytotoxicity induced by carboplatin and Olaparib [32].

Kopper et al. discovered this correlation by testing homologous recombination in a subset of organoid lines with different responses to Niraparib using the recombination capacity (RECAP) test, which assesses homologous recombination capacity using the accumulation of RAD51 protein at sites of DNA double-strand breaks 49 and geminin as a marker for the S/G2 phases of the cell cycle. Organoids with a low percentage of geminin positive cells with RAD51 foci were more sensitive to Niraparib compared with organoids with a high percentage of geminin-positive cells with RAD51 foci. This observation suggests that OC PDOs could be used to assess the HRD status independently of the mutational signature, thus potentially uncovering defects in genes and pathways not yet associated with HR [35]. Another research team devised 7 PDO lines covering 3 subtypes (HGSOC, CCC and CE) for drug sensitivity and resistance testing [34]. Organoids possessing the BRCA1 pathogenic variant (p.L63*) were more sensitive to Olaparib and platinum drugs, while those originating from the clear-cell subtype were resistant to conventional chemodrugs [34]. Tao et al. demonstrated OC PDOs’ capacity to evaluate the sensitivity to PARPi under different settings, exploring the mechanisms of resistance, and identifying effective combined strategies, which have implications for the clinical application of PARPi [44].

Another important innovative possibility of PDOs is to test the immune checkpoint inhibitors (ICI). Wan et al. compared the action of innovative, bispecific, anti-PD-1/PD-L1 ICB antibodies to monospecific anti-PD-1 and anti-PD-L1 molecules. This study showed that the bispecific antibody uniquely induces state changes in NK cells from inert to active states and most strongly induces a state change in CD8 T cells from naive to cytotoxic progenitor exhausted states [45,46].

## 5. Clinical Implications and Future Prospective

### 5.1. Patient Derived Organoids (PDOs)

**Figure 1:**
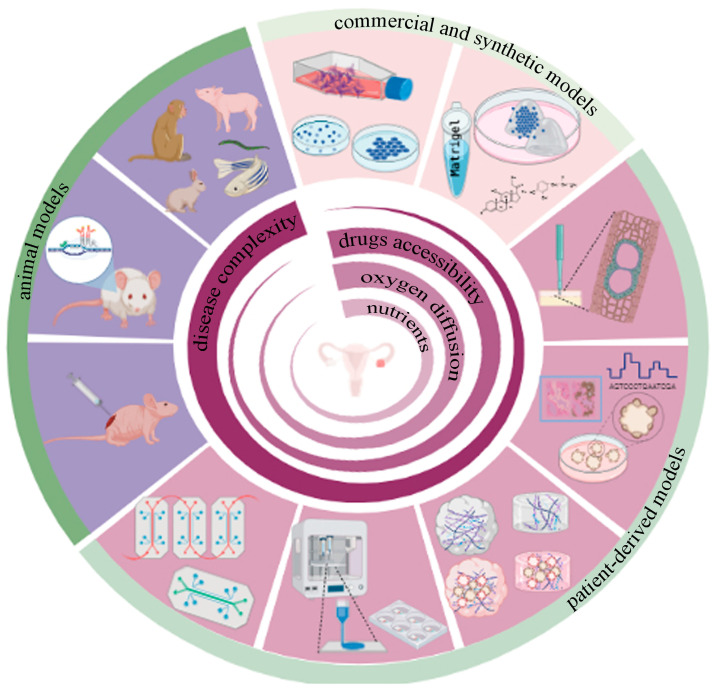
Preclinical models for epithelial ovarian cancer: clockwise, in light pink, commercial and synthetic preclinical models that include stabilized cells growing in conventional plastic plates, cell-derived spheroids, and synthetic hydrogels repopulated with cells or spheroids. The patient-derived preclinical model’s shown in dark pink represent ex-vivo tumor slices, PDOs, decellularized ECM (dECM) and hydrogel-derived, either dECM alone or repopulated with PDO, 3D bioprinting technology, and organ on a chip based on microfluidics technology, aiming to mimic not only ovarian tumors but also the metastatic sites. Purple denotes preclinical animal models including PDX, GEMM, and other animal species commonly used in scientific research, especially at the later stages of clinical trials.

PDOs are becoming an important and powerful preclinical model for personalized medicine. In recent decades, there has been growing interest in tailoring cancer therapy to each patient (Figure 1). Often, when we refer to precision medicine, we indirectly refer to genomics [55]. However, as Anthony Letai masterfully explained in the review published in *Nature Medicine*, only a limited proportion of patients have an actionable alteration that can be associated with a specific drug intervention [56]. Functional precision medicine—based on testing drug responses in patient tumors to identify treatment regimens—has been proposed as a more robust alternative. As described by Friedman et al., PDOs provide an unprecedented opportunity to improve preclinical drug discovery, clinical trial validation and, ultimately, patient care because they are simple to establish, have high similarity to native tissue, and are easy to treat, as demonstrated in different types of tumors, including ovarian cancer [57]. Undoubtedly, reproducibility is an essential aspect of obtaining solid data about treatment response that can be translated into clinic scenarios. Intra- and interorganoid heterogeneity limit reproducability in the organoid model. Intraorganoid heterogeneity is the variability between cells forming the organoid, whereas interorganoid heterogeneity is the variability between organoids in the same dish and between individual patients [55]. In particular, although there are a variety of OC subtypes, high-grade serous carcinoma, low-grade serous carcinoma, clear cell carcinoma, mucinous carcinoma, and endometrioid carcinoma, these are treated as a single disease. In this context, the ability of the ovarian PDO model to mimic the complexity of the pathology is a great advantage in carrying out specific drug screenings [35]. In 2019 Kopper et al. established 56 organoid lines from 32 patients, representing all main subtypes of OC. OC organoids recapitulate the histological and genomic characteristics of the native lesion from which they were derived, illustrating intra- and inter-patient heterogeneity [35].

Undoubtedly, over the years, immortalized cell lines, spheroids, PDXs and GEMMs have represented the gold standards of scientific pre-clinical models (Figure 1) [58]. However, they fail to represent the real complex of tumor biology; in fact, all models presented some limitations. For example, the process of immortalizing primary tissues cell lines is very inefficient, and extensive genetic shifts occurred during propagations [59]. PDXs do not fully recapitulate tumor heterogeneity all the time; the human TME cannot be preserved because the original stroma is mostly replaced by murine-derived fibroblasts and the extracellular matrix. Second, PDX is not a suitable candidate for testing immunotherapies. To avoid rejection after grafting foreign tumor cells, PDX models require the use of immunocompromised mice that lack NK, B and T cells, so these mice cannot recapitulate actual immune responses in humans [60]. The mutations induced in GEMMS to produce disease phenotypes do not precisely capture the diversity of human disease phenotypes or subtypes. In addition, GEMMS are generally seen as poor predictors of clinical success [61].

Another important aspect is represented by the opportunity offered by PDOs to create a comprehensive biobank of well-characterized healthy and diseased patient-derived tissue. For example, at Christie Hospital in Manchester, UK, from 2016 to 2019, 312 samples were collected from patients with chemo-naive epithelial ovarian cancer and relapsed disease. Thanks to Nelson L. et al., we were able to study typical HGSOC chromosome instability [62]. Another example of ovarian PDO biobank was reported by de Witte et al. between January 2016 and September 2019 at the University Medical Centre Utrecht and Leiden University Medical Centre, the Netherlands, where they collected epithelial ovarian cancer samples with the aim of comparing the clinical response of PDO drugs. Patient data and tissue collection were carried out according to the guidelines of the European Network of Research Ethics Committees [41]. Comprehensive research on PDO-related clinical trials has been noted in ClinicalTrials.gov, but only one piece of research focused on ovarian cancer. Despite the promising features of organoids, their utility is balanced by a variety of limitations. They are unable to mirror structures of the TME, such as the surrounding mesenchyme, blood vessels, and immune cells. Moreover, PDOs lack vascularization and, consequentially, the diffusion of oxygen and nutrients is limited.

### 5.2. Tumor Microenvironment (TME) and Extracellular Matrix (ECM)

At present, it is not reasonable to consider cancer a cellular-only disease. From this point of view, thinking of PDO as a model that completely reassembles the patient’s neoplasm is limiting. The TME plays a structural function but, more importantly, contributes to tumor evolution and remodeling. Stromal dysregulation, as well as cell proliferation, is consistent with neoplastic progression [63]. From tumor initiation to metastasis, intricate and reciprocal interactions between ovarian cancer cells and the stromal components of their surrounding milieu create a complex and fluctuating TME [64].

Stromal components of the TME include the tumor vasculature and lymphatics (including endothelial cells and pericytes), mesothelial cells, fibroblasts, immune cells, and ECM proteins.

In the primary tumor niche, cancer cells recruit cancer-associated fibroblasts, T-cells, endothelial cells and tumor-associated macrophages. Many components of ECM, such as fibronectin, hyaluronan, tenascin, versican, matrix metalloproteinases (MMPs) and lysyl oxidase (LOX), are upregulated, while essential components such as collagen I and IV progressively decrease and are remodelled into randomly orientated thin fibres. Mechanical forces such as shear stress and stiffness caused by increased peritoneal fluid flow also contribute to this environment, inducing changes in cell morphology and gene expression. All these elements are associated with specific features of tumorigenesis and metastasis, and their involvement cannot be accurately captured by traditional 2D cell culture systems [65].

Taking into consideration the importance of mimicking the TME, the model most used in laboratories today is that, following PDOs isolation, they are typically seeded into biologically derived matrices such as Matrigel or a natural extracellular matrix such as a mix of collages. Matrigel, a mouse sarcoma extracellular matrix protein mixture, is an indispensable component of most organoid tissue cultures [66]. However, the utility of organoids for drug development has been limited due to its tumor-derived origin, batch-to-batch variation, high cost, and safety issues [55]. Furthermore, for Matrigel, as well as other gels of commercial origin, the poorly defined composition offers little control over the biochemical and biophysical spatio-temporal signals that are required to enhance the PDO culture as a robust preclinical tool.

In 1985, Niedbala et al. were the first to establish an organotypic culture of ovarian cancer TME and investigate the mechanism through which ovarian cancer cells infiltrate the mesothelial cell layer and attach to the ECM [67]. This model allows for an examination of the role that ECM and fibroblasts play in the initial adhesion, migration, invasion, and proliferation of ovarian cancer cells during early metastases to the mesothelium. In this context, it is easy to understand the fundamental role that further investigations of the extracellular matrix in ovarian cancer can play.

There are two main ways to overcome the use of nonspecific ECM: one is the use of synthetic matrices with more complete control over composition and mechanical properties, and the other is to consider the tissue decellularization technique to create tissue-specific matrices [68,69].

Decellularization means the removal of the cellular component of a tissue by minimally altering its biochemical composition and biological and structural properties. In the review by Mendibil et al., a complete overview of the most common decellularization methods was provided [70]. Organs such as the heart [71], lung [72], liver [73], colon [63], pancreas [74], diaphragm [75] were successfully decellularized. Undoubtedly, the principal advantage of using biological-derived ECM instead of synthetic polymers for the 3D in vitro tumor study is that a portion of the main structural proteins and soluble factors are already present in the decellularized scaffolds. For this reason, decellularization has recently been applied to unravel the complex and fundamental role of ECM in tumor progression, inflammation, and metastasis.

At present, there are no reports using decellularized ECM (dECM) from the ovary for the study of ovarian cancers. A handful of studies have shown high biocompatibility with ovarian cell types grown in reconstituted dECM hydrogels and scaffolds. A mixture of synthetic polymer with decellularized murine ovarian tissue supports the survival of the in vitro follicle [76]. Some time ago, Kim et al. presented a paper in which they described the generation of ECM-hydrogels derived from gastrointestinal patients that allowed for the long-term culturing of organoids by providing tissue-mimetic microenvironments (Figure 1) [66].

There are no analogous manuscripts in gynecological oncology, except a study of Buckenmeyer et al. in which hydrogels derived from decellularized porcine ovarian ECM underlined the effect of ECM stiffness on ovarian follicle development, with stiffer matrices reducing oocyte viability and triggering premature follicle release, but no evidence is present in the literature using OC cells. Although we believe that the dECM is a great preclinical too, there are some concerns about the is use, namely donor batch differences, the retention of native genetic material, and the ddifficult decellularization process that can result in the loss of critical downstream biological interactions with cells [55]. Three-dimensional bioprinting presents a solution that combines ECM components and the high-throughput creation of models of ovarian cancer in a spatially controlled manner. As this is a relatively new approach to 3D cell model creation, the number of published studies for ovarian cancer is limited. The droplet-based technique was used by Xu et al. for bioprinting drugs, growth factors, OC cells, and normal fibroblasts on top of a Matrigel scaffold to study the interaction between tumor and stromal cells [77]. To date, there have been no publications utilizing drop-on-demand inkjet bioprinting, laser-assisted bioprinting, or stereolithography with ovarian cancer cells. In 2022, T Wu et al. used 3D printing to design and fabricate a 3D artificial ovary using an extrusion-based method with gelatin-methacryloyl bioink [78]. They demonstrated its valid application in the treatment of female endocrine and reproductive conditions.

Finally, we believe that combining patient-derived dECM and PDOs using 3D bioprinting has great potential as a future basis for preclinical studies guiding drug development and screening for ovarian cancer treatment.

### 5.3. Drug Screening

Given the high rate of advanced stage at diagnosis and recurrence of ovarian cancers, there is a clear need for more models that can predict drug response, both at diagnosis and during relapse, to guide the therapeutic strategy.

In fact, cancer therapy is rapidly progressing toward individualized regimens based on the molecular characteristics of tumors and not on the organ of origin.

Three trials are currently being carried out to evaluate the role of PDOs in predicting the clinical efficacy of anticancer drugs (chemotherapy and targeted therapy) in OC (NCT04279509, NCT04768270 and NCT04555473).

The NCT04279509 trial drug screen assays using PDOs can accurately select chemotherapeutic agents, resulting in an objective response in patients with refractory solid tumors (head and neck squamous cell carcinoma, colorectal, breast, and epithelial OC). NCT04768270 is a single-centre study that aims to verify whether PDOs can help guide precision treatments for OC patients. NCT04555473 is a phase II longitudinal observational study of the reliability of HGSOC PDOs as a model for patients’ response to treatments.

According to current evidence, one of the main problems in the long-term management of OC patients is the high rate of relapse and the development of drug resistance. Given this, the possibility of improving OC therapy in patients with recurrence using predictive PDO is an important topic of interest.

In the near future, the ability to perform drug screening using PDO models for efficacy and resistance mechanisms with both traditional and new molecular targeted therapy and drug combinations (i.e., PARPi and ICI) has the potential to boost personalized medicines. In fact, some authors have investigated PDOs’ capacity to evaluate the sensitivity of EOCs to PARPi. This has been one of the most successful examples of precision medicine to date, exploring potential resistance mechanisms, and identifying effective combined strategies, all of which have implications for the clinical application of PARPi [31,32,34,35,41,44].

Furthermore, including stromal, immune, and vascular cells in these cultures will have important clinical implications, allowing for the better recapitulation of the patient’s environment. This will be particularly relevant for HGSOC, where several targeted therapies involve angiogenic inhibitors and immune checkpoint inhibitors (ICI). The efficacy of these agents depends on many factors, including PD-L1 expression, the abundance of tumor-infiltrating lymphocytes (TILs), neoantigen load, and the tumor mutational burden. The initial overoptimism regarding the use of these agents in HGSOC treatment has been tempered by the disappointing results that emerged in clinical trials [79,80,81].

The main goals will be to use this organoid for targeted drug research and to study combination therapy, comparing the response in PDO derived from PDS, prior diagnostic biopsy neoadjuvant chemotherapy (NACT) therapy and interval debulking surgery (IDS) in order to influence future clinical decisions. Furthermore, they will allow for drug testings with similar or identical targets applied to different subtypes of organoids to study their interaction mechanism and identify the combination with the best efficiency and fewest side effects.

## 6. Conclusions

Considering the high mortality rate of patients with OC, an in vitro, 3D model of the disease is essential to determine personalized therapy. In this way, PDOs represent reliable experimental models that recapitulate the molecular characteristics and heterogeneity of the original tumor. They may contribute important knowledge tfor the prediction of treatment response, particularly in the context of NACT therapy and the course of recurrent disease. In fact, clinical diagnostic molecular approaches such as next-generation sequencing are being used to develop biologically targeted therapies that could be used to guide treatment decisions based on the response to PDO.

However, the current major problems with their use to support clinical decisions are related to their success rate, development time, and the integration of TME for better drug screening. To increase the formation and define the development time, it is crucial to standardize the known procedures of tissue manipulation, the media, and the growth factors required to improve the success rate and drug screening. At the same time, to provide a good platform for drug screening, the complexity of organoids must be increased by incorporating TME (vascular system and immune cells) and personalized ECM.

With further improvements, the OC organoid could become a promising and efficient method for the primary organ culturing of gynecological cancer, as well as a highly reliable research strategy for testing combination therapy, targeted therapy, and immunotherapy research aiming to select a therapy for the patient based on PDO response.

## Figures and Tables

**Table 1:** Patient-derived organoids (PDOs).

	Site of Origin	Histological Types	Number of Patients	Number of Organoids	Success Rate	Genomic Characterization	Concordance Rate
Pauli 2017 [30]	Ovaries	Serous	1	1	100%	WES	86% concordance between organoids and native tumor tissues (based on the analysis of 1062 specific cancer genes).
Jabs 2017 [31]	Ovaries Ascites Pleural effusion	HGSC	9	9 (8 + 1)	100%	WGS	n.s
Hill 2018 [32]	Ovaries Omentum Pleural effusion Mesentery Diaphragm	HGSC LGSC	22	33	80–90%	WES	98.8% of mutations were identified both in the tumors and in the matched organoid line.
Phan 2019 [33]	Ovaries Peritoneum	Peritoneal HGSC Carcinosarcoma	4	4	100%	Not performed	n.s
Kopper 2019 [35]	Ovaries Peritoneum Diaphragm Rectum Lymph nodes Ascites Pleural effusion	EC BOT HGSC LGSC MC CCC	32	56	65%	WGS	High in S-SNVs and CNVs.
Maru 2019 [36]	n.s	EC Brenner tumors HGSC MC CCC	15	9	90%	409 gene panel	High
Hoffman 2020 [37]	Peritoneum Omentum	HGSC	13	15	30%	121 gene panel	High
Sun 2020 [38]	n.s	Serous	10	10	n.s	RNA-seq	n.s
Maenhoudt 2020 [40]	Ovaries Omentum Rectum	HGSC LGSC CCC MC MT	27	12	44%	WGS	98% of the genetic alterations (S-CNAs) similarly present in both primary tumor and resultant organoid line.
Nanki 2020 [34]	Ovaries	HGSC CCC EC BOT non-serous OC	35	28	80%	1053-gene panel	Median concordance 59.1% (36.1–73.1%) of the genomic variants were shared among organoids and primary tumours
de Witte 2020 [41]	Ascites Omentum Adnexa Peritoneum Lymph nodes Uterus	HGSC EC LGSC CCC BOT MC	23	36	n.s	WGS	67% of single-nucleotide variants, comparable copy-number states
Chen 2020 [42]	Ascites Pleural effusion	HGSC Peritoneal HGSC	6	14	n.s	RNA-seq	n.s
Gorski 2021 [43]	n.s	HGSC	6	6	100%	DNA-seq RNA-seq	High
Tao 2022 [44]	Primary tumor and metastatic lesions	HGSC EC CC BOT	n.s	7	85%	WES	91.5% of SNVs and SVs present in the original tumor were maintained in the derived organoids.
Wan 2021 [45]	n.s	HGSC	13	12	92%	RNA-seq	n.s
Wang 2022 [39]	n.s	HGSC	n.s	n.s	n.s	RNA-seq	n.s
Wan 2022 [46]	Ovaries	BOT	13	10	77%	RNA-seq	High

Legend: Whole-exome sequencing: WES; whole-genome sequencing: WGS; somatic single-nucleotide variants: S-SNVs; structural variants: SVs; somatic copy number alterations: S-CNAs; copy number variations: CNVs; RNA sequencing: RNA-seq; high-grade serous carcinoma: HGSC; low-grade serous carcinoma: LGSC; endometrioid carcinoma: EC; ovarian cancer: OC; mucinous carcinomas: MC; clear-cell carcinoma: CCC; borderline ovarian tumor: BOT; mesonephric tumor: MT; not specified: n.s.

**Table 2:** Extracellular matrix (ECM).

	Extracellular Matrix	Culturing Medium	Organoid Formation (Days)
Pauli 2017 [30]	Matrigel	Glutamax, B27 (Gibco), 100 U/mL penicillin, 100 μg/mL streptomycin, Primocin 100 μg/mL, N-Acetylcysteine 1.25 mM, Mouse Recombinant EGF 50 ng/mL, Recombinant Human FGF-basic 1 ng/mL, Y-27632 10 μM, A-83-01 500 nM, SB202190 10 μM, Nicotinamide 10 mM, PGE2 1 μM, Noggin 50 mL, R-Spondin 25 mL	n.s
Jabs 2017 [31]	2% Matrigel	50 lg/mL gentamicin, 0.5 lg/mL Fungizone, lM ROCK inhibitor Y27632, 5% CO_2_	Up to 10 days
Hill 2018 [32]	Matrigel	Advanced DMEM/F12, 1% penicillin streptomycin, Glutamax, 1% HEPES, 100 ng/mL R-spondin 1, 100 ng/mL Noggin, 50 ng/mL EGF, 10 ng/mL FGF-10, 10 ng/mL FGF2-1× B27, 10 mM Nicotinamide, 1.25 mM N-acetylcysteine, 1 μM Prostaglandin E2, 10 μM SB202190, 500 nm A83–01-Y-27632	7–14 days
Phan 2019 [33]	Matrigel or Cultrex BME	PrEGM medium or Mammocult	n.s
Kopper 2019 [35]	Cultrex BME	25% conditioned human RSPO1 medium, 12 mM HEPES, 1% Glutamax, 2% B27, 10 ng mL^−1^ human EGF, 100 ng mL^−1^ human noggin, 100 ng mL^−1^ human FGF10, 1% N2 10 mM nicotinamide, 9 μM ROCK inhibitor, 0.5 μM, TGF, βR Kinase Inhibitor IV, hydrocortison, forskolin, heregulinβ-1	3–14 days
Maru 2019 [36]	Matrigel	DMEM/F12 (Thermo Fisher Scientific, Watham, MA, USA), 50 ng/mL human EGF (Peprotech, Rocky Hill, NJ, USA), 250 ng/mL R-spondin1 (R&D, Minneapolis, MN, USA), 100 ng/mL Noggin (Peprotech), 10 μM Y27632 (Wako, Osaka, Japan), 1 μM Jagged-1 (AnaSpec, Fremont, CA, USA), L-glutamine solution (Wako), penicillin/streptomycin (Sigma-Aldrich, St. Louis, MO, USA), amphotericin B suspension (Wako)	n.s
Hoffman 2020 [37]	Matrigel	Wnt3a (mouse 25%), R-Spondin 1 (mouse 25%), FGF 10, human 100 ng·mL^−1^, Noggin, human 100 ng·mL^−1^, EGF, human 10 ng·mL^−1^,Y-27632 9 µM, SB431542 0.5 µM, B27 supplement 1x, N2 supplement 1x, Nicotinamide 1 mM, GlutaMax 100x 1x Hepes 10 mM, Penicillin/Streptomycin 100 U·mL^−1^/100 mg·mL^−1^, Advanced DMEM/F12 1x	n.s
Sun 2020 [38]	Matrigel	n.s	n.s
Maenhoudt 2020 [40]	Matrigel	DMEM/F12 (Thermo Fisher Scientific), 10% fetal bovine serum (FBS; Thermo Fisher Scientific), 2% Penicillin/streptomycin, 10% dimethyl sulfoxide, 1–5 mM Nicotinamide, 1.25 mM N-acetylcysteine, 10 nM 17-β Estradiol, 10^−1^ μM p38i (SB203580), 2 ng/mL bFGF (OCOM 1-2), 50 ng/mL NRG1 (OCOM4), 10 ng/mL FGF10 (OCOM1-2), 50 ng/mL RSPO1 (rec or CM), 20 ng/mL IGF1 (OCOM3-4)	n.s
Nanki 2020 [34]	Matrigel	Advanced DMEM/F12, 2mMHEPES, 1 × GlutaMAX-I, 1X B27 supplement 10 nM Leu15-Gastrin, 1 mM N-acetylcystein, 100 ng/mL recombinant human IGF-1, 50 ng/mL recombinant human FGF-2, 20% Afamin/Wnt3a CM, 1 μg/mL humanR-spondin, 100 ng/mL Noggin, 500 nM A-83-01, 200 U/mL penicillin/ streptomycin 10 μM Y-27632	7–21 days
de Witte 2020 [41]	Matrigel	1% GlutaMAX, 2%B27, 1%N2, 10 ng mL^−1^ human EGF, 100 ng mL^−1^ human noggin, 100 ng mL^−1^ human FGF10, 1 mM nicotinamide, 9 μM ROCK inhibitor, 0.5 μMTGF-βR Kinase Inhibitor IV, Hydrocortisone, Forskolin, heregulinβ-1	20 days
Chen 2020 [42]	Cultrex Reduced Growth Factor Basement Membrane Extract, Type 2 (BME)	DMEM/ F12, 10% R-spondin1 2% B27 Supplement, 10 mM HEPES, 1% Glutamax, 1.25 mM N-acetyl cysteine, 100 μg/mL Primocin, 1% Antibiotic-Antimycotic, 1 mM nicotinamide, 0.5 μM A 83–01, 5 nM Neuregulin 1, 5 ng/mL FGF-7, 20 ng/mL FGF-10, 100 ng/mL Noggin, 5 ng/mL EGF-0.5 μM, SB 202190 5 μM, Y-27632	3–4 days
Gorski 2021 [43]	Matrigel	n.s	n.s
Tao 2022 [44]	Matrigel	Oded Kopper’s Protocol, Advanced DMEM/F12, 1x Glutamax, 10 mM HEPES, Noggin, Rspo1, N-Acetylcysteine (500 mM), Primocin, A83-01 (5 mM), Fgf10 (100 μg/mL), Heregulinβ-1 (75 μg/mL), Y27632 (100 mM), EGF (500 μg/mL), Forskolin (10 mM), Hydrocortisone (250 μg/mL), β-Estradiol (100 μM)	n.s
Wan 2021 [45]	15% Matrigel	DMEM/10% FBS, 1% Pen/Strep, 30 ng/mL of IL-2	n.s
Wang 2022 [39]	Matrigel	DMEM, 1% penicillin-streptomycin, 10 mmol/L nicotinamide	7 days
Wan 2022 [46]	70% Matrigel	Oded Kopper’s Protocol, Advanced DMEM/F12, 1x Glutamax, 10 mM HEPES, Noggin, Rspo1, N-Acetylcysteine (500 mM), Primocin, A83-01 (5 mM), Fgf10 (100 μg/mL), Heregulinβ-1 (75 μg/mL), Y27632 (100 mM), EGF (500 μg/mL), Forskolin (10 mM), Hydrocortisone (250 μg/mL), β-Estradiol (100 μM)	n.s

Legend: not specified: n.s.

**Table 3:** Drug screening.

	Traditional Anticancer Drugs	PARPi/ICI	Others (Action Mechanism)
Pauli 2017 [30]	Not performed		
Jabs 2017 [31]	Carboplatin, Paclitaxel, Doxorubicin	Olaparib	MK5108 (Aurora A kinase inhibitor), NSC23766 (inhibitor of Rac1 activation), Cyclopamine (inhibitor of the Hh pathway), AZD2014 (inhibitor of mTORC1 and mTORC2), AZD5363 (Akt inhibitor), BKM120 (PI3Ki), Decitabine, Azacytidine, Belinostat (HDACi), Dasatinib (TKI)
Hill 2018 [32]	Carboplatin	Olaparib	Prexasertib (CHK1 inhibitor), VE-822 (ATR inhibitor)
Phan 2019 [33]			240 protein kinase inhibitor compounds FDA-approved or in clinical development at two various concentrations
Kopper 2019 [35]	Carboplatin, Paclitaxel, Gemcitabine	Niraparib	Alpelisib, Pictilisib, MK2206, AZD8055 (PI3K/AKT/mTOR pathway inhibitors), Adavosertib (Wee1 inhibitors)
Maru 2019 [36]	Paclitaxel, Cisplatin		
Hoffman 2020 [37]	Carboplatin		
Sun 2020 [38]	Cisplatin		
Maenhoudt 2020 [40]	Paclitaxel, Doxorubicin, Carboplatin, Gemcitabine		Nutlin-3 (MDM2 inhibitors)
Nanki 2020 [34]	Cisplatin, Carboplatin, Paclitaxel, Docetaxel, Vinorelbine, Doxorubicin, Gemcitabine, Tamoxifen, Trabectedin	Olaparib	Vorinostat (HDACi), Belinostat (HDACi), Cediranib (VEGFi), Pazopanib (VEGFi), Topotecan, Eribulin, SN-38, Etoposide
de Witte 2020 [41]	Carboplatin, Paclitaxel, Gemcitabine	Olaparib Niraparib Rucaparib	Afatinib (EGFR inhibitor), Vemurafenib (B-Raf Inhibitor), Flavopiridol (CDK inhibitor), Adavosertib (Wee1 Inhibitors), Alpelisib (PISKi), AZD8055 (PISKi), Pictilisib (PISKi), Cobimetinib (MEKi)
Chen 2020 [42]	Carboplatin, Taxol		Mocetinostat 8 (HDAC inhibitor), Trametinib (MEK inhibitor), LY294002 (PI3k inhibitor), AZD5363 (Akt Inhibitor), BBI503 (NANOG/CD133 Inhibitor), MK-1775 (Wee-1 Inhibitor), APR-246 (p53 reactivator), CB5083 (ATPase inhibitor), Napabucasin, (STAT3 Inhibitor), Sorafenib (VEGFi)
Gorski 2021 [43]	Carboplatin		
Tao 2022 [44]	Cisplatin, Paclitaxel, Gemcitabine	Niraparib Olaparib	AZD7762 (Chk1 inhibitor), SAHA (HDACi), deguelin, SN-38 (topoisomerase I inhibitor),
Wan 2021 [45]		bispecific anti-PD-1/PD-L1 ICB antibodies to monospecific anti-PD-1 and anti-PD-L1 molecules	
Wang 2022 [39]	Not performed		
Wan 2022 [46]			Bractoppin (BRCA inhibitor)

Legend: Poly (ADPribose) polymerase (PARP) inhibitors: PARPi; Immune check point inhibitors: ICI; Tyrosine kinase inhibitors: TKI.
